# Description of neurotoxicity in a series of patients treated with CAR T-cell therapy

**DOI:** 10.1038/s41598-020-76055-9

**Published:** 2020-11-04

**Authors:** Catherine Belin, Perrine Devic, Xavier Ayrignac, Amélie Dos Santos, Adrien Paix, Lila Sirven-Villaros, Claire Simard, Sylvain Lamure, Thomas Gastinne, Renata Ursu, Colette Berger, Laura Platon, Benoît Tessoulin, Elie Azoulay, Florent Wallet, Catherine Thieblemont, Emmanuel Bachy, Guillaume Cartron, David A. Laplaud, Antoine F. Carpentier

**Affiliations:** 1 Department of Neurology, Hôpital Saint-Louis, Assistance Publique - Hôpitaux de Paris, 1 Avenue Claude Vellefaux, 75010 Paris, France; 2 Department of Clinical and Functional Neurology, Hospices Civils de Lyon, CHU Lyon Sud, 165 Chemin du Grand Revoyet, 69495 Pierre-Bénite, France; 3 Department of Neurology, Centre Hospitalier Universitaire de Montpellier, INSERM, 80 Avenue Augustin Fliche, 34295 Montpellier, France; 4 Department of Neurology, CRTI-InsermU1064, CIC 1413, Centre Hospitalier Universitaire de Nantes, 5 Allée de l’île Gloriette, 44093 Nantes, France; 5 Institut de Radiothérapie de Bobigny, Ramsay Générale de Santé, Rue Lautréamont, 93000 Bobigny, France; 6 Université de Paris, Paris-Diderot, 75010 Paris, France; 7 Department of Clinical Haematology, Centre Hospitalier Universitaire de Montpellier, 80 Avenue Augustin Fliche, 34295 Montpellier, France; 8 Department of Clinical Haematology, Centre Hospitalier Universitaire de Nantes, 5 Allée de l’île gloriette, 44093 Nantes, France; 9 Intensive Care Medicine Department, Lapeyronie Hospital, Centre Hospitalier Universitaire de Montpellier, 80 Avenue Augustin Fliche, 34295 Montpellier, France; 10 Intensive Care Medicine Department, Hôpital Saint-Louis, Assistance Publique-Hôpitaux de Paris, 75010 Paris, France; 11 Intensive Care Medicine Department, Hospices Civils de Lyon, CHU Lyon Sud, 165 Chemin du Grand Revoyet, 69495 Pierre-Bénite, France; 12 Department of Haemato-Oncology, Hôpital Saint-Louis, Assistance Publique-Hôpitaux de Paris, 75010 Paris, France; 13 Department of Clinical Haematology, Hospices Civils de Lyon, CHU Lyon Sud, 165 Chemin du Grand Revoyet, 69495 Pierre-Bénite, France; 14 INSERM U1052 CNRS, UMR5286, Centre de Recherche en Cancérologie de Lyon &, Université Claude Bernard, Lyon 1, France

**Keywords:** Lymphoma, Neurotoxicity syndromes

## Abstract

Chimeric antigen receptor-modified T (CAR T) cell therapy is a highly promising treatment for haematological malignancies but is frequently associated with cytokine release syndrome and neurotoxicity. Between July 2018 and July 2019, all patients treated with CD19-targeted CAR T-cell therapy for relapsing lymphoma were followed-up longitudinally to describe neurological symptoms and their evolution over time. Four different French centres participated and 84 patients (median age 59 years, 31% females) were included. Neurotoxicity, defined as the presence of at least one neurological symptom appearing after treatment infusion, was reported in 43% of the patients. The median time to onset was 7 days after infusion with a median duration of 6 days. More than half of the patients (64%) had grade 1–2 severity and 34% had grade 3–4. CRS was observed in 80% of all patients. The most frequent neurological symptoms were cognitive signs, being severe in 36%, and were equally distributed between language disorders and cognitive disorders without language impairment. Non-pyramidal motor disorders, severe in 11%, were reported in 42% of the patients. Elevation of C-reactive protein (CRP) within 4 days after treatment was significantly correlated with the occurrence of grade 3–4 neurotoxicity. Although sometimes severe, neurotoxicity was almost always reversible. The efficacy of steroids and antiepileptic drugs remains unproven in the management of neurotoxicity. Neurotoxicity associated with CAR T-cell therapies occurs in more than 40% of patients. The clinical pattern is heterogeneous but cognitive disorders (not limited to language disorders) and, to a minor degree, non-pyramidal motor disorders, appeared as a signature of severe neurotoxicity.

## Introduction

Chimeric antigen receptor-modified T (CAR T) cells have emerged as a promising therapeutic strategy for haematological malignancies. Multiple clinical trials of CAR T cells have demonstrated high rates of clinical responses and durable remissions in B-cell hematologic malignancies leading to the recent approval of two different CAR Ts (tisagenlecleucel and axicabtagene ciloleucel) in diffuse large B cell lymphoma (DLBCL), B cell-precursor acute lymphoblastic leukaemia (ALL), and in large cell transformation of follicular lymphoma (trFL). The impressive efficacy of CAR T-cells, however, is associated with significant and sometimes life-threatening toxicities, the most prevalent being cytokine release syndrome (CRS) and neurotoxicity (NTX)^1–4^.

CRS results from generalized immune activation and correlates with dramatic elevations of inflammatory cytokines including interferon gamma, granulocyte macrophage colony-stimulating factor, or IL-6^4^. CRS is extremely frequent, with percentage ranging from 60 to 93%, but only 13–14% for grade 3 and 4^5^. Clinical features of CRS include high fever, fatigue, myalgia, nausea, disseminated intravascular coagulation that can progress to life-threatening vasodilatory shock, capillary leak, hypoxia, and end-organ dysfunction^6^. Both systemic corticosteroid and IL-6 receptor (IL-6R) blockade (mainly tocilizumab) can rapidly reverse CRS symptoms and are commonly prescribed as front-line treatments for CRS. However, the impact of prolonged use (e.g. > 2 weeks) of high-dose corticosteroids on CAR T-cell proliferation, persistence, and antitumour effect remains unclear^7^.

NTX is recognized as a different entity, described as CAR T-Cell Related Encephalopathy (CRES) by Neelapu et al.^8^ and recently under the name of ICANS^9^ (Immune effector Cell–Associated Neurotoxicity Syndrome). Neurologic symptoms typically occur after CRS with variable delay. Their clinical spectrum is very heterogeneous, making it difficult to identify the most relevant symptoms. In the literature, patients are described as variably suffering from encephalopathy, confusional state, aphasia, myoclonus, or other central nervous system disorders^2,3,10–12^. In an attempt to homogenize clinical practice, the American Society for Transplantation and Cellular Therapy (ASTCT, formerly American Society for Blood and Marrow Transplantation, ASBMT) has proposed a specific grading scale for ICANS^9^.

The precise physiopathology of NTX remains unclear. Blood brain barrier dysfunction within the nervous system has been suggested as a key mechanism, as brain oedema has been reported in a few patients^13^. Yet, most patients experienced severe neurotoxicity without any abnormality on brain imaging^3^. In a nonhuman primate model, neurotoxicity was associated with elevated inflammatory cytokines in the cerebrospinal fluid (CSF), as well as both CAR and non-CAR T-cell recruitment^14^. As a consequence of this largely unknown physiopathology, there is no consensus on the most effective therapeutic interventions to mitigate neurologic toxicity of CAR T-cells^4,5,8,12,15,16^.

We herein report the neurological symptoms experienced by the first 84 consecutive patients undergoing CAR T-cell therapy, outside clinical trials, in four different centres in France from July 2018 to July 2019, with the purpose to better characterize the incidence, clinical spectrum and evolutive pattern of neurological symptoms.

## Methods

Between July 1st 2018 and July 1st 2019, all patients treated with commercially produced CD19-targeted CAR T-cell therapy for relapsing B-cell lymphoma, were prospectively identified and monitored for signs of neurotoxicity. Patients treated within clinical trials were excluded from this study, in order to have results in real life outside of clinical trials. Four different centres (Paris/Saint-Louis, Lyon/CHU Lyon Sud, Montpellier and Nantes) participated in this study. Informed consent have been obtained from all participants in this study. This study was approved by the local institutional review board for ethics and clinical research “Comité Local d’Ethique pour la Recherche Clinique des HUPSSD Avicenne-Jean Verdier-René Muret” (CLEA-2019-74) and conducted in compliance with STROBE Statement^17^.

### Toxicity assessment

Several grading systems have been proposed for the evaluation of toxicity after CAR T-cell therapy^8,18^, as recently emphasized by Pennisi et al.^19^. In our study, two different scales were used: the National Cancer Institute Common Terminology Criteria for Adverse Events (CTCAEv_5)^20^, and the American Society for Transplantation and Cellular Therapy (ASTCT) consensus grading for toxicity associated with immune effector cells^9^. CRS grading^6^ was assigned by the inpatient attending haematologist or intensivist. For neurological assessments, all patients were followed prospectively from the time of admission and examined by a neurologist before infusion, and 7 and 14 days after infusion of CAR T-cells. In case of neurological symptoms, patients were examined by the neurologist within 24 h, and then regularly assessed until neurologic symptom resolution. All neurological symptoms documented in clinical notes and discharge summaries from CAR T-cells infusion up to 2 months post treatment were reviewed. NTX grades according to CTCAEv_5 and ASTCT were assigned by the inpatient attending neurologist and reviewed retrospectively by two neurologists (CB and AC). Discrepancies were adjudicated by consensus review with the attending neurologist.

### Statistical analysis

For statistical analysis, continuous variables are presented as mean ± standard deviation (SD) in normally distributed variables and as median in non-parametrical variables. Qualitative variables are presented as absolute and relative frequencies. Missing data, when inferior to 5%, were imputed using the predictive mean matching (PMM) method^21^. Factors associated with neurotoxicity were assessed with a multivariate logistic regression model. To select the variables included in the model, we assessed the potential multicollinearity between variables with the Variation Inflation Factor (VIF) and used a threshold of 4. Factors associated with NTX were first evaluated with a univariate logistic regression with a significant cut-off of *p* < 0.2. A stepwise backward procedure was used to build the multivariate logistic regression to evaluate the potential factors associated with neurotoxicity. Results of the multivariate regression model are expressed as Odds Ratio (OR) with 95% confidence interval and a *p* value with a significant threshold of *0·05*. Statistical analyses were conduct using R software version 3.6.0. Data can be accessed by contacting the authors.

### Ethics approval and consent to participate

This study was approved by the local institutional review board for ethics and clinical research “Comité Local d’Ethique pour la Recherche Clinique des HUPSSD Avicenne-Jean Verdier-René Muret” (CLEA-2019-74) and conducted in compliance with STROBE Statement.

## Results

### Patients’ characteristics

Eighty-four consecutive patients receiving CAR T-cell treatments for B-cell lymphomas (76 diffuse large B-cell lymphoma, six transformed follicular lymphoma, and two follicular lymphoma) were prospectively followed (n = 44, 18, 13 and 9 patients in Paris, Lyon, Montpellier, and Nantes centres, respectively). All patients in this series were adults; ages ranged from 19 to 77 years old and the median age was 59. Twenty-six patients (31%) were female. Before CAR T-cell infusion, neurological examination was considered as normal in 59 patients (70%). Twenty patients (24%) had a peripheral sensitive neuropathy due to previous chemotherapies. Two patients had Horner syndrome related to lymphoma localization. One patient had a history of Wallenberg syndrome with sequellae. One patient showed mild executive problems and another one mild mnesic impairment (Mattis Dementia Rating Scale^22^ score of 125/144 six months before infusion).

In two of the four centres, all patients (n = 57) underwent systematic cerebral imagery before treatment (MRI in 56 patients and CT-scan for one claustrophobic patient). These baseline cerebral imagings showed abnormalities in six cases: two cases of meningiomas, one vascular leucopathy, one venous developmental anomaly, and two patients had lymphomatous cerebral localization. In the 51 other cases, MRIs were considered normal with respect to the age of patient, 22% having non-specific rare white matter abnormalities.

All patients were treated with CAR T-cells: Tisagenlecleucel in 51 pts (61%) and Axicabtasene Ciloleucel in 33 pts (39%).

### Incidence of CRS and NTX

Sixty-seven patients (80%) had CRS (grade 1–2 in 63 patients (94%), grade 3–4 in 4 patients (6%)). Median day for CRS onset was day 3 and median duration of CRS was five days.

**Figure 1:**
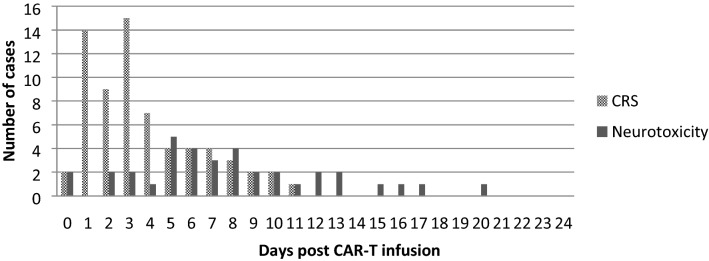
Day of onset for CRS and neurotoxicity.

Thirty six patients (43%) developed neurological symptoms: Grade 1–2 CTCAEv5.0 in 23 patients (64%), and grade 3–4 in 13 patients (36%). Median day of onset of neurological symptoms was day 7 (range 0 – 20) (Fig. 1), and median duration of NTX was 6 days. NTX was associated to CRS in 100% of cases (grade 1–2 CRS in 32/36 patients; grade 3–4 CRS in 4/36 patients).

### Clinical description of NTX & outcomes

The neurological symptoms observed in the 36 patients were classified into five categories: (1) cognitive signs, (2) non-pyramidal motor disorders, (3) consciousness disorders, (4) seizures, and (5) miscellaneous (Table 1).

**Table 1:** Neurotoxicities: distribution of neurological signs within the five categories.

Neurological signs	n	Categories	n
Aphasia	10	Cognitive signs	43
Executive syndrome	7		
Agraphia	5		
Cognitive slowness	5		
Confusional state	4		
Apraxia	2		
Disorientation	2		
Restlessness	2		
Attentional disorders	1		
Dysarthria	1		
Dyscalculia	1		
Hallucination	1		
Memory disorders	1		
Neglect syndrome	1		
Cerebellar syndrome	7	Non-pyramidal motor disorders	16
Myoclonus	4		
Tremor	3		
Asterixis	1		
Dyskinesia	1		
Consciousness disorders	6	Consciousness disorders	5
Seizures	3	Seizures	3
Headaches	5	Miscellaneous	9
Dysesthesias	2		
Meningismus	1		
Transitory focal weakness	1		

The “cognitive signs” category contains not only aphasic (n = 10) and agraphic signs (n = 5) but also executive disorders (n = 7), cognitive slowness (n = 5), apraxia (either ideomotor or ideatory n = 2), disorientation (n = 2), restlessness (n = 2), and attentional disorders, dysarthria, dyscalculia, hemineglect syndrome (n = 1 for each). In this category, hallucinations (one case of sensory hallucination) and confusionnal states (n = 4) were also included. Each of these signs could be isolated (e.g. pure anomia) or associated.

The “non-pyramidal motor disorders” category gathers all types of non-pyramidal motor signs i.e. movement disorders and cerebellar signs. The most frequent symptoms combine static and kinetic cerebellar disorders (seven patients). We also observed four cases of myoclonus, three cases of upper limb postural tremor, one case of asterixis and one case of upper limb dyskinesia.

The “consciousness disorders” category refers to altered consciousness, ranging from somnolence to comatose states. In this series, only mild alteration *i.e.* somnolence and/or depressed level of arousal was reported in six patients.

The “seizure category”, in our series, includes three cases of tonic–clonic generalized seizures. No focal epileptic syndrome was reported.

The “miscellaneous category” brings together the signs that did not fit in the other categories: headaches in five patients, one case of meningismus, two patients with dysesthesia and one patient who had a transitory focal brachio-facial deficit.

The two patients with pre-existing cerebral lymphomatous localization (before CAR T-cells infusion) did not have NTX related to treatment. One of these patients had an important increase of its intra-cerebral lymphomatous mass at day 7 leading to death at day 60.

**Figure 2:**
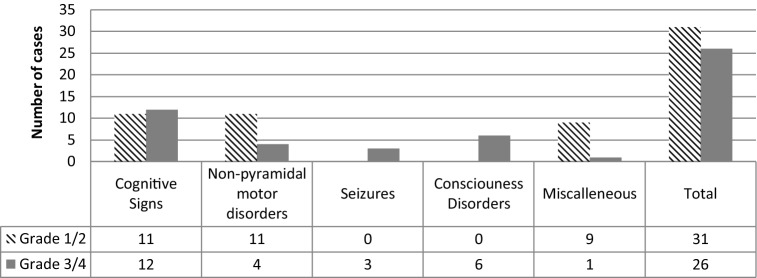
Neurotoxicities: number of patients per category and grade (patients can belong to several categories).

CTACE Grade 1 and 2 neurotoxicities (23/84 patients; 27%) were mostly described as mild cognitive disorders (n = 11), non-pyramidal motor disorders (n = 11), or headaches (n = 6). For CTCAE grade 3 and 4 neurotoxicities (13/84 patients; 15%), all patients but one had cognitive disorders, either isolated (n = 1) or associated with one other category (non-pyramidal motor disorders n = 2, consciousness disorders n = 4, seizures n = 3) or with two other categories (non-pyramidal motor disorders and consciousness disorders, n = 2; dysesthesia, n = 1). The only patient who was not described as having cognitive signs had consciousness disorders and asterixis (Fig. 2).

### Concordance between CTCAE and ASTCT grading scale

In half of the patients (18/36), the CTCAEv5.0 and ASTCT grading scales were concordant (three grade 1, four grade 2, eight grade 3 and three grade 4). In the 18 non-concordant patients, eleven grade 1 and one grade 2 CTCAE signs were not captured by the ASTCT grading scale which excludes certain neurological signs (headache, tremors, abnormal movements, hallucinations). Four patients with grade 2 CTCAE were graded 1 in ASTCT scale. In the other two patients, the discrepancies were significant (grade 3 CTCAE versus grade 1 ASTCT), mostly because the ASTCTscale does not take into account cognitive signs other than those affecting language (such as hemineglect syndrome or executive disorders) or cerebellar signs.

### Evolution and treatment

Within two months after reinfusion, 11 patients (13%) died, four of them before day 21. The median age of these patients was 58 years (23–70), comparable to the median age of the entire cohort (59 years). In all patients, the cause of death was attributed by the attending clinicians to progression of the disease, associated to septic complications in three cases. Among these eleven patients, five had neurological signs, but the role of NTX in death is unclear because of the concomitant CRS, tumour progression and/or sepsis in these patients.

All other patients (87%) recovered from NTX within two months after reinfusion. The median duration time of NTX was 6 days (1–47). The median duration time of grade 1–2 NTX was 6 days (1–17) and grade 3–4 NTX was 5 days (2–47).

Thirty-five patients with CRS (52%) were treated with IL-6 receptor blocker (tocilizumab).

Twenty patients with NTX received steroid treatment (methylprednisolone in three cases, dexamethasone in 17 cases) with a treatment duration ranging from 1 to 33 days (median of 8 days). Among the 13 patients with a grade 3–4 NTX, all but one received steroids. In the 23 patients who had a grade 1–2 NTX, eight (35%) received steroids. The median duration time of NTX was 8 versus 5 days in steroids treated versus non steroids treated patients respectively (Table 2).

**Table 2:** Evolution of grade 1–2 neurotoxicity (NTX) depending on steroid treatment.

	Treatment with steroid	No treatment with steroid
Number of patients with grade 1/2 NTX	8	15
median duration of NTX (days)	**8** (3–17)	**5** (1–9)
number of days with steroids	10 (1–22)	NA

Twenty-two patients received anti-epileptic drugs: five patients had long-term treatments (pregabaline, gabapentine, diazepam) for neuropathic pains and seven patients were treated preventively with levetiracetam, starting before (four cases) or on the day of infusion (three patients). Six patients started a prophylactic treatment on the first day of NTX, although there was no clinical sign of seizure or epileptic activity on EEG, and three patients were treated with levetiracetam after tonico-clonic generalized seizures. Despite preventive anti-epileptic treatment with levetiracetam in seven patients, three developed NTX.

### Paraclinical investigations

Among the 36 patients who developed NTX, 27 underwent brain imaging: 21 magnetic resonance imaging (MRI) and 10 computerized tomography (CT) scan. All these radiological examinations were normal or identical to the MRI performed before the CAR T-cell infusion.

CSF study, performed in 11 patients, was abnormal in nine patients; two had isolated high protein level, two had isolated pleiocytosis, and five had both hyperproteinorachia and pleiocytosis. Protein level varied from 0·53 g/L to 6·33 g/L (median 1·04 g/L) and white blood cells count from 6/mm^3^ to 171/mm^3^ (median 19/mm^3^). In one case, high CSF pressure (30 cm H_2_O) was reported. CSF cultures and viral serologies were negative in all cases.

Nineteen patients underwent electroencephalograms (EEG). Only one patient had epileptiform discharges, while the others had mostly generalized slowing background in the theta-delta range**.**

### Predictive factors

We then analysed the association between various factors and the occurrence of severe (Grade 3–4) NTX (Table 3). Because Cefepim can be neurotoxic^23^, treatment of CRS with this antibiotic was also considered.

**Table 3:** Characteristics of patients without or with grade 3–4 neurotoxicity (NTX).

	No NTX	Grade 3–4 NTX
Number of patients	48	13
Age (mean ± SD)	53 ± 14	57 ± 17
% Female	31%	38%
Tisagenlecleucel/Axicabtasene Ciloleucel	23/25	3/10
Pre-treatment CRP in mg/L (median; IQR)	10; 38	33; 59
Occurrence of CRS (%) median CRS grade	65% 1	100% 2
Highest CRP within 4 days after reinjection in mg/L (median; IQR)	34; 60	114; 155
Lowest platelet count within 4 days after reinjection (median; IQR)	102; 80	83; 102
Highest ferritin within 4 days after reinjection (median; IQR);	841; 1016	1996; 3334
Treatment with Cefepim (%)	13%	31%

In univariate analysis, only post- treatment C-reactive protein (CRP) and ferritin levels showed a significant association with NTX (Table 4). In multivariate analysis, only post-treatment CRP remained significantly associated with neurotoxicity (*p* = 0.047), post-treament ferritinemia being borderline (*p* = 0.051). The type of CAR T-cells did not show any predictive value on the occurrence of NTX.

**Table 4:** Logistic regression model of potential factors on occurrence of grade 3–4 neurotoxicity (univariate analysis).

	Odds ratio	IC 95%		*p*
		Inf	Sup	
**Sex**				
Female	Ref			
Male	0.72	0.21	2.75	0.62
Age	1.02	0.98	1.07	0.45
**CAR T-cells**				
AC	Ref			
T	0.33	0.07	1.22	0.12
Pre-treatment CRP (median in mg/L)	1.01	1.0006	1.0286	0.057
CRP max (median in mg/L)	1.008	1.002	1.015	0.01
Highest ferritin after reinjection (median in µg/L)	1.0005	1.0002	1.001	0.006
Lowest platelet count within 4 days after reinjection (median)	0.998	0.991	1.003	0.35
Treatment with Cefepim	3.1	0.68	13.4	0.13

## Discussion

Here, we report the first, multicentre French series of patients treated with CAR T-cells for B-cell malignancies outside clinical trials. Neurological disorders occurred in 43% of our patients (severe in one third of the cases), with a median time to onset of seven days after infusion and a median duration of symptoms of six days, in line with previous reports^2,3^. The most striking feature in CAR T-cells induced NTX is its highly heterogeneous spectrum, however limited to the central nervous system. In the literature, encephalopathy is the most frequently reported symptom^1–3,7,10,11,16^, but this nosological entity encompasses a rather large spectrum of signs or symptoms and thus does not help the referring physicians in their daily practice. In this series, all patients experiencing NTX were examined by a neurologist, and the described neurological symptoms mainly fall into two categories: cognitive impairment (23/36 patients; 64%) and non-pyramidal motor disorders (15/36 patients; 42%).

Cognitive disorders were reported in all of our patients with grade 3–4 NTX, except one who was had asterixis and consciousness disorders important enough to make it difficult to assess cognitive impairment. Cognitive disorders are sometimes lacking in grade 1–2 neurological signs (12/23 pts; 52%), but in these non-serious events, mild cognitive deficits might have been unnoticed without a detailed neuropsychological assessment. It should be stressed that cognitive signs are not limited to language disorders. Among the 23 patients with cognitive disorders of all grades, language disorders were present in only 12 patients (52%), either isolated (26%), or associated with other cognitive disorders (26%). Interestingly, the other half of the patients had cognitive impairment without any language disorder: executive and frontal behavioural disorders, ideomotor and ideational apraxia, dyscalculia, hemispatial neglect. The figures are similar if we consider the patients with grades 3–4 NTX only: seven patients (58%) showed language disorders (isolated in 3 cases, associated with other cognitive disorders in four patients) and five patients (42%) had cognitive impairment without any language impairment. The other series frequently report language disorders^2,3,11^, with particular emphasis on writing disorders^8^, but exceptionally reports other types of cognitive impairment as did Rubin et al.^3^ who reported a limited subset of patients with isolated apraxia. This point is significant because only language disorders are routinely investigated by clinicians following the recommendations of the consensus ASTCT which promote grading of NTX based on the CARTOX^8^ or ICE^9^ scales. Routine examinations in treated patients should therefore not be limited to speech impairment, and should also include evaluation of executive, praxic, gnosic, spatial or mnesic functions. Additional neuropsychological test exploring the different facets of cognition, such as the MoCa test^24^ should be part of the systematic clinical workup, both before CAR T-cells infusion and whenever neurological disorders are suspected.

Non-pyramidal motor disorders were observed in 4/13 patients (30%) with grade 3–4 NTX. Cerebellar syndromes were included in this category, because distinction between myoclonus, tremor and kinetic cerebellar syndrome can be challenging in some patients. As already noted by Rubin et al.^3^, asterixis, myoclonus and tremor are often the earliest sign of NTX, and were found in 11% of the patients in our series. Similarly, cerebellar disorders (any grade) were found in 8%. In that respect, it looks surprising that these frequent symptoms are excluded from grading scale of ASTCT Consensus^9^, even if movement disorders are usually mild.

Seizures (tonic–clonic generalized seizures in our series) were reported in 3/84 patients (3·5%), and epileptiform discharges was observed in 1/19 EEG (5%). This incidence, in line with other series^3,11^, might be underestimated by the occurrence of asymptomatic partial seizures. However, the fact that some patients developed NTX under preventive treatment with levetiracetam raises the question of the large prescription of this drug in a preventive setting. Interestingly, myoclonus was observed in some patients and could be, in theory, considered as a pre-epileptic symptom. In one patient, we had the chance to monitor by EEG a patient with myoclonus, but no specific cortical activity was recorded.

Headaches are frequently reported in the literature (up to 55% in the series of Gust et al.^11^), but were seen in only 7% of our patients. Headaches are usually non severe. This point is of interest because alteration of the blood brain barrier is a putative mechanism for NTX^11^. Yet, we did not observed any patient experiencing severe headaches leading to suspect intracranial hypertension.

With rare exceptions (dysesthesias linked to meralgia paresthetica, C2 radicular neuralgia, isolated aphasia, upper limb transitory deficit), none of the disorders observed in our patients can be explained by a single focal lesion. In accordance with this finding, brain MRI made in 85% of our patients with a grade 3–4 NTX did not provide any argument in favour of focal lesions. This is in line with others studies^3,7^, in which conventional MRI was not the best paraclinical investigation to highlight neurological dysfunctions. Functional imaging by PET-FDG scan is probably more effective in showing metabolic rather than structural anomalies, as Rubin et al.^3^ showed in six of their patients.

Identifying predictive factors for NTX would be particularly useful for early management of patients at risk. Various factors associated with NTX have been proposed in other series^2–4,11,16^, some being related to the patient’s history (high pre-treatment tumour burden, low baseline platelet count, high baseline CRP), and others to the way the patient is responding to treatment (occurrence of CRS, CAR T-cell expansion, fever on day 3). Some biological markers after CAR T-cells infusions have also been suggested, such as CRP or ferritin. However, many of these factors can be biased with conditioning chemotherapy and/or concomitant infections. In our series, baseline platelet count, baseline and post-treatment CRP, severity of CRS, fever on day 3, post treatment ferritin, type of CAR T-cells and treatment with Cefepim were tested in multivariate analysis. Only the elevation of CRP within four days after treatment was significantly correlated with the occurrence of grade 3–4 NTX. We acknowledge that this statistical analysis should be taken with caution, given the limited number of patients included in this series.

Almost all patients recovered from NTX within two months after reinfusion. Eleven patients (13%) died, but in all these cases the role of NTX in the fatal outcome was either unlikely, or questionable. The median duration time of NTX was 6 days, comparable to literature^1–3,11^. Steroids are nowadays widely prescribed in case of NTX, but this was not the case in 2018. This shift in the standard of care allowed us to compare the evolution of patients whether treated or not with steroids. Surprisingly, the impact of steroids on the duration of neurological symptoms was not conclusive (8 versus 5 days in steroid-treated versus non steroid-treated patients, respectively). This figure should be taken with caution as this retrospective analysis on a limited number of patients is subjected to bias. However, this observation strongly supports the need for a more evidence-based prescription of steroids in these patients, as steroids carry a significant morbidity, and can perhaps jeopardize the efficacy of CAR T-cells at high doses^7^.

In conclusion, the pattern of NTX in this homogeneous group of patients treated for B-cell lymphoma identified cognitive disorders and, to a minor degree, non-pyramidal motor disorders, as the hallmark of NTX linked to CAR T-cell therapy. These symptoms are insufficiently captured with available scales, which should be updated accordingly. Although sometimes severe, neurotoxicity is almost always reversible. The efficacy of steroids and antiepileptic drugs remain unproven and should be further evaluated in prospective trials.

## Data Availability

Data are available from the corresponding author on reasonable request.

## Abbreviations

ALL: Acute lymphoblastic leukaemia
ASBMT: American Society for Blood and Marrow Transplantation
ASTCT: American Society for Transplantation and Cellular Therapy
CAR: Chimeric antigen receptor-modified
CRES: CAR T-cell related encephalopathy
CRP: C-reactive protein
CRS: Cytokine release syndrome
CTCAE: Common terminology criteria for adverse events
CT: Computerized tomography
CSF: Cerebro-spinal fluid
DLBCL: Diffuse large B cell lymphoma
EEG: Electroencephalogram
ICANS: Immune effector cell–associated neurotoxicity syndrome
MRI: Magnetic resonance imaging
NTX: Neurotoxicity
OR: Odds ratio
PMM: Predictive mean matching
SD: Standard deviation
trFL: Transformation of follicular lymphoma
VIF: Variation inflation factor
